# Airway microbiota correlated with pulmonary exacerbation in primary ciliary dyskinesia patients

**DOI:** 10.1128/spectrum.02213-23

**Published:** 2023-10-05

**Authors:** Weitao Zhou, Zhuoyao Guo, Jinglong Chen, Yao Chen, Chen He, Aizhen Lu, Liling Qian

**Affiliations:** 1 Department of Respiratory Medicine, Children’s Hospital of Fudan University, Shanghai, China; McGill University, Ste-Anne-de-Bellevue, Quebec, Canada

**Keywords:** airway microbiota, pulmonary exacerbation, primary ciliary dyskinesia, microbiota diversity

## Abstract

Primary ciliary dyskinesia (PCD) is characterized by symptoms such as productive cough, rhinitis, and recurrent infections in both the upper and lower airways, which can lead to chronic infection, bronchiectasis, and decreased pulmonary function. Our study aimed to analyze the diversity and composition of the microbiota in the respiratory tract and investigate the correlation of the microbiota with pulmonary exacerbation in PCD patients. This study employed 16S rRNA amplicon sequencing to explore the microbiota present in both bronchoalveolar lavage fluid (BALF) and sputum samples collected from PCD patients. Eighty-five airway samples (17 BALF and 68 sputum samples) were collected from 72 patients aged 2 months to 60 years. Significantly lower sputum microbiota diversity and richness were found in the pulmonary exacerbation group than in the pulmonary stabilization group; additionally, the relative abundance of *Pseudomonas* increased when pulmonary exacerbation occurred. There was a positive correlation between pulmonary exacerbation and the relative abundance of *Pseudomonas*, while *Streptococcus*, *Moraxella*, and *Haemophilus* showed a negative correlation. Although there was no significant difference in the sputum microbiota diversity and composition in children aged 6–18 years with pulmonary exacerbation, *Pseudomonas* increased during pulmonary exacerbation. BALF samples of pediatric patients with pulmonary exacerbation had a lower microbiota diversity and richness; furthermore, *Pseudomonas* had a higher abundance and a moderate distinguishing effect. We found that microbiota beta diversity and dominance were decreased during pulmonary exacerbation. Additionally, *Pseudomonas* had a higher abundance and moderate distinguishing effect in pediatric patients with pulmonary exacerbation.

**IMPORTANCE:**

PCD is a rare disease characterized by productive cough, rhinitis, and recurrent infections of the upper and lower airways. Because the diagnosis of PCD is often delayed, patients receive more antibiotics, experience a heavier financial burden, and have a worse prognosis; thus, it is very important to identify the pathogeny and use the correct antibiotic. In this large single-center study of PCD microbiota, we identified an outline of the bacterial microbes from the respiratory tract; furthermore, we found that the microbiota diversity in pediatric sputum was richer than that in pediatric BALF through sequencing, indicating a heterogeneous community structure. The microbiota diversity and richness were lower during pulmonary exacerbation than during pulmonary stabilization. A significantly higher abundance of *Pseudomonas* had a moderate distinguishing effect for lung exacerbation, which attracted more attention for the study of *Pseudomonas* therapy in pediatric patients with PCD.

## INTRODUCTION

Primary ciliary dyskinesia (PCD) is characterized by productive cough, rhinitis, and recurrent infections of the upper and lower airways. Situs inversus occurs in approximately 40%–50% of patients; other disease manifestations associated with PCD include infertility in males, hydrocephalus, and complex heart disease (1). PCD is a rare disease, and the predicted worldwide morbidity varies between 1:2,000 and 1:40,000 according to different methods (2). The diagnosis of PCD is difficult due to its clinical variability and extensive genetic heterogeneity. Notably, mutations that lead to this syndrome have been discovered in approximately 50 genes, and newly related genes continue to be reported (3).

As the diagnosis of PCD is often delayed, patients receive more antibiotics, bear a heavier financial burden, and have a worse prognosis (4). Occult PCD patients are affected in early childhood and exhibit pulmonary exacerbations and variable prognoses, so it is crucial to precisely diagnose and distinguish the stable stage (SS) from the exacerbated stage (SE). Chronic infections play a role in pulmonary exacerbation, which leads to bronchiectasis and high mortality (5). Several microbes, such as *Pseudomonas aeruginosa*, *Haemophilus influenzae*, *Staphylococcus aureus*, *Moraxella catarrhalis*, and *Streptococcus pneumoniae*, have been discovered in PCD patients by traditional culture methods (6). *H. influenzae* is the dominant bacteria in children, and *P. aeruginosa* infection in teenagers and adults is a common cause of reduplicative lung infections, chronic infection, bronchiectasis, and decreased pulmonary function (7). However, in addition to these traditionally cultured microbes, the microbiota of PCD patients comprises unculturable microbes.

Due to the development of next-generation sequencing, 16S rRNA gene amplicon sequencing can be used to identify detailed airway bacteria in pulmonary diseases (8). The diversity of the human lower respiratory airway microbiome increases gradually before reaching stability within the first 2 postnatal months, after which the composition resembles the healthy adult lung microbiota (9). Cystic fibrosis (CF) patients of different ages have been shown to have different microbial susceptibilities and diverse prognoses (10). In patients with bronchiectasis, the risk of exacerbation was found to be associated with reduced complex microbial cooccurrence networks, decreased diversity, and a higher prevalence of antagonistic interactions in the airway microbiome; furthermore, interactions within the *Pseudomonas* microbial community enhance the clinical predictability of exacerbation models (11). To date, only two studies have reported a positive correlation between bacterial load and age and no significant difference between community diversity and pulmonary function in PCD patients (12, 13).

In this study, 16S rRNA amplicon sequencing was used to analyze collected bronchoalveolar lavage fluid (BALF) and sputum samples from PCD patients. We hypothesized that the airway microbiota diversity would differ between BALF and sputum and that some dominant genera may correlate with pulmonary exacerbation.

## MATERIALS AND METHODS

### Study design and patients

A retrospective cross-sectional study was conducted including 72 PCD patients during a 3-year period (July 2020 to December 2022) at the Children’s Hospital of Fudan University. Seventeen BALF samples and 49 sputum samples from children with PCD were collected at the hospital, and 19 sputum samples from adult patients were transferred on ice to the laboratory from the patients’ homes. All samples were collected in the first 3 days of illness. These samples were immediately placed in a −80°C refrigerator once they arrived at the laboratory. The study was approved by the ethics committee of the Children’s Hospital of Fudan University.

### PCD diagnosis and BMI

PCD was diagnosed by the international diagnostic guidelines as published previously (14). Pulmonary exacerbation was diagnosed by the expert consensus definition from 2019, which stated that patients should meet three or more criteria (15). Pulmonary function was measured by the European Respiratory Society/American Thoracic Society (ERS/ATS) guidelines (16), and forced expiratory volume in 1 second (FEV1) was chosen as the pulmonary function indicator. According to the American Medical Association guidelines from 2005, pulmonary ventilatory disorders were classified into three levels. Age-adjusted body mass index (BMI) z scores in PCD patients were calculated according to the guidelines of the World Health Organization (WHO). There were no BMI z scores for adults, which were computed based on the reference values for 19-year-olds (17).

### 16S rRNA gene library preparation and sequencing

Total DNA from sputum and BALF samples was extracted using the sodium dodecyl sulfate (SDS) method and purified with a TIANgel Purification Kit (Qiagen, Germany) (18). The DNA concentration was measured with Qbit 4, which was followed by 250 bp double-ended sequencing of the V3 and V4 regions on an Illumina MiSeq 300 (19). The sequencing data underwent refinement to remove adaptors, low-quality reads, and ambiguous bases. After removing chimeric sequences using DADA2, the amplicon sequence variants (ASVs) were classified based on 99% similarity, and a representative sequence was obtained from each ASV cluster. The alpha diversity was calculated using the Shannon index; the beta diversity was assessed using Bray‒Curtis distances in QIIME and visualized by principal coordinate analysis (PCoA). The random forest R package was used to analyze a random forest classifier model trained by 10-fold cross-validation. The Wilcoxon rank sum test was used to compare taxonomic relative abundance between the two groups.

### Statistical analysis

SPSS22 software and R version 4.0.2 were used for statistical analysis of all data. Age range, z score, FEV1, and nasal nitric oxide are expressed as the median (quartile spacing: Q1 and Q3) with a skewed distribution. The Mann‒Whitney *U* test was used to compare the differences in Shannon index and microbe mean relative abundance between the two groups. PCoA plots were created to visualize the sample separation using pairwise distances, and permutational multivariate analysis of variance (PERMANOVA) was conducted to assess the statistical significance of the differences observed in PCoA. Receiver operating characteristic curves were used to analyze the correlation between the microbiota abundance at the genus level and pulmonary exacerbation. Spearman’s test was used to measure the relationship between FEV1 and age. Statistical significance was determined by two-tailed *P* values less than 0.05.

## RESULTS

### Population clinical characteristics

From July 2020 to December 2022, 72 patients were recruited, 34 males and 38 females with a median age of 11.3 years (range: 6.7–22.8). Fifteen patients were younger than 6 years old, with a median age of 2.3 years (range: 1.3–4.5); 38 patients were aged 6–18 years, with a mean age of 11.2 years (range: 8.7–12.8); and 19 patients were adults, with a median age of 30 years (range: 28–38). Seventeen BALF samples and 68 sputum samples were collected, and the clinical data are summarized in Table 1; Table S1. The average BMI z score was −1.35 (range: −0.41 to 0.40), which had no correlation with age. The mean FEV1 of 53 patients was 70% (range: 52.7%–84.1%); 16 people had an FEV1 >80% (mean 87.9%; 95.4–93.5); 20 people had an FEV1 between 60% and 80% (mean 70.1%; 65.3%–74.2%); eight people had an FEV1 between 40% and 60% (mean 52.7%; 51%–56.2%); and nine people had an FEV1 less than 40% (mean 35.8%; 31%–37.6%). FEV1 had a significant negative correlation with age (r = −0.536, *P* < 0.05), which was consistent with a previous study (7). The average nasal nitric oxide level of 38 people was 12.2 (range 8.7–25). At the time of sampling, there were 43 individuals with a stable condition, 46 patients were undergoing antibiotic therapy, and 45 patients had bronchiectasis. All participants had complications of wet cough and sinusitis.

**TABLE 1:** Summary characteristics of the study population^*a*^

Subject characteristics	Data
Age range (median)	11.3 y (6.7, 22.8)/72
<6 y [% (no.)]	2.3 (1.3, 4.5)/15
6–18 y [% (no.)]	11.2 (8.7, 12.8)/38
>18 y [% (no.)]	30 (28, 38)/19
Male/female ratio [% (no.)]	47.2/34
Z score	1.35 (−0.41, 0.40)
FEV1% pred [% (no.)]	70 (52.7, 84.1)/53
>80	87.9 (85.4, 93.5)/16
60–80	70.1 (65.3, 74.2)/20
40–60	52.7 (51, 56.2)/8
<40	35.8 (31, 37.6)/9
Nasal nitric oxide	12.2 (8.7, 25)/38
Stable at time of sampling (no.）	43
Antibiotic therapy at time of sampling (no.)	45
Wet cough	72
Sinusitis	72
Bronchiectasis	46

^*a*^ FEV1= forced expiratory volume in 1 second.

### Correlation of pulmonary exacerbation with sputum microbiota

**Fig 1:**
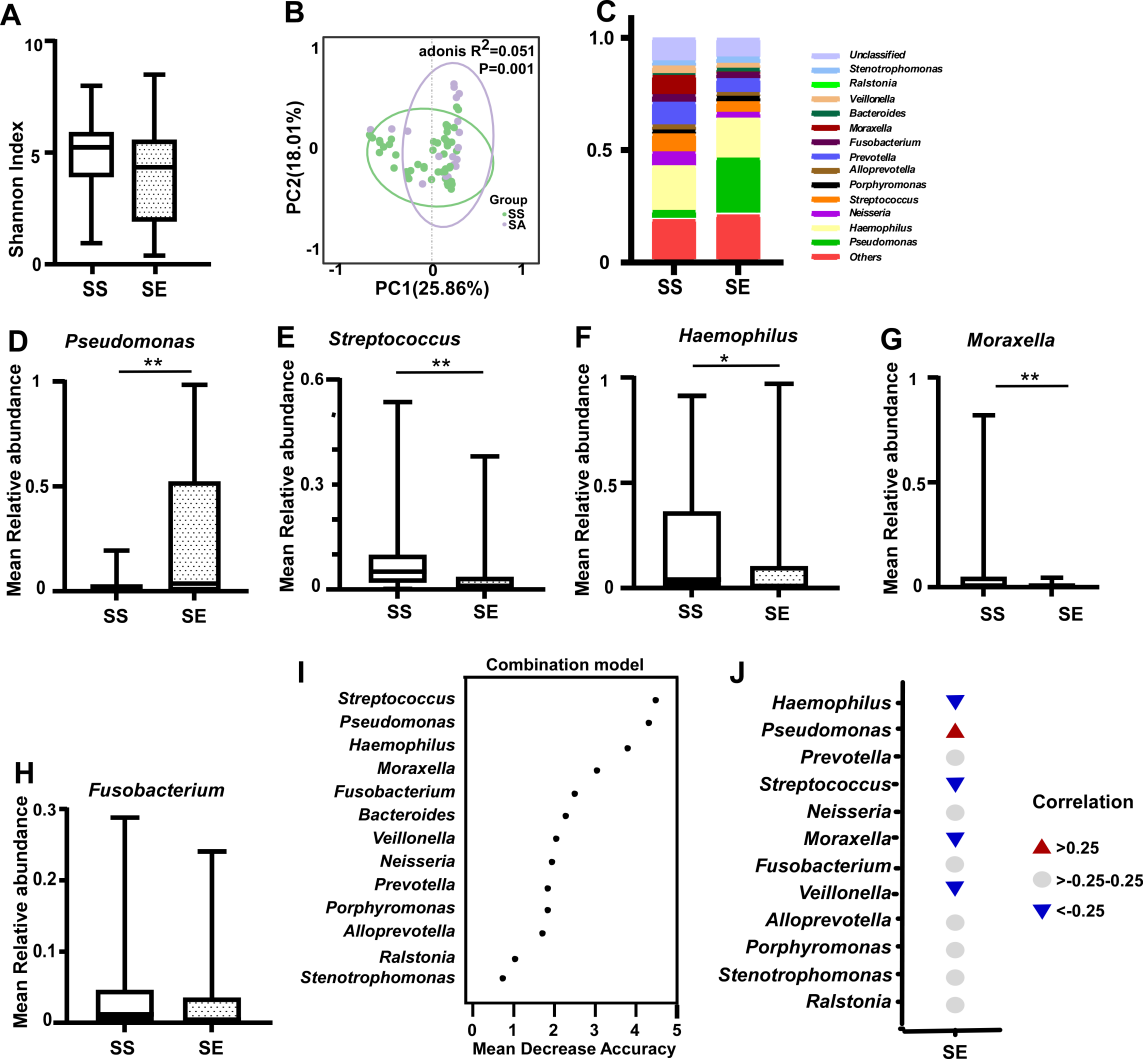
Structural shift in the sputum microbiota in patients with PCD. (A) Microbial diversity in the SS and SE groups (Shannon index; *P* > 0.05). (B) Principal coordinate analysis (PCoA) based on weighted UniFrac distance by analysis of similarities (Adonis; *P* < 0.01). (C) The relative abundance of microbial taxa at the genus level; genera with a relative abundance ranking in the top 1%. (D–H) Median relative abundance for *Pseudomonas* (D), *Streptococcus* (E), *Haemophilus* (F), *Moraxella* (G), and *Fusobacterium* (H) associated with SS and SE PCD patients. (I) Classification performance of the top 1% most discriminant genera of the SE group by a random forest model. (J) Spearman’s correlation coefficients for the relative abundance of bacterial taxa related to sputum exacerbation. Red, positive correlations; blue, negative correlations; gray, no correlations. Positive (up) and negative (down) correlations are also indicated by the direction of the arrowhead symbol in the heatmap. Correlation values >0.25 or <−0.25 have *P* values < 0.05. Boxes show the 25th–75th interquartile range (IQR). The median is indicated by a solid line in the box. PCD: primary ciliary dyskinesia, SS: sputum in stable stage (*n* = 41), SE: sputum in exacerbation stage (*n* = 27), *: *P* < 0.05; **: *P* < 0.01 for statistically significant differences.

Filtered reads through 16S sequencing in all samples were between 41,115 and 133,092, and the ASV number ranged from 36 to 1805. First, we investigated the relationship between pulmonary exacerbation and the sputum microbiota in all 68 samples. The Shannon index (Table S2), an alpha-diversity index, was used to describe sample bacterial richness and diversity. The Shannon index of sputum in the SS was lower than that of sputum in the SE, but the difference was not significant (Fig. 1A). PCoA was performed with calculated weighted UniFrac distance metrics, which reflected bacterial diversity among different groups (Table S3), and PCoA significantly differed between the SS and SE groups (PERMANOVA: *P* = 0.001; *R* ^2^ = 0.056; Fig. 1B). The microbiota compositional nonuniformity (Table S3) was different between the SS group and SE group (Fig. 1C). The relative abundance of *Pseudomonas* was significantly higher during pulmonary exacerbation (Fig. 1D); however, the relative abundances of *Haemophilus*, *Streptococcus*, and *Moraxella* were significantly lower (Fig. 1E through G). *Fusobacterium* was lower in the SE group, but there was no significant difference (Fig. 1H). A selected model of SE was established using the top 1% of abundant genera by random forest analysis, which can be used to distinguish the key microbial taxonomy in different groups, and *Streptococcus*, *Haemophilus*, and *Pseudomonas* were the top three significantly different genera (Fig. 1I). There was a positive correlation between pulmonary exacerbation and the relative abundance of *Pseudomonas* (*R* = 0.312, *P* = 0.0095), while *Streptococcus* (*R* = −0.364, *P* = 0.0023), *Moraxella* (*R* = −0.334, *P* = 0.0053), and *Haemophilus* (*R* = −0.281, *P* = 0.020) showed a negative correlation (Fig. 1J).

### Association between pulmonary exacerbation and the sputum microbiota dominated by *Streptococcus* and *Moraxella* in school-aged children

**Fig 2:**
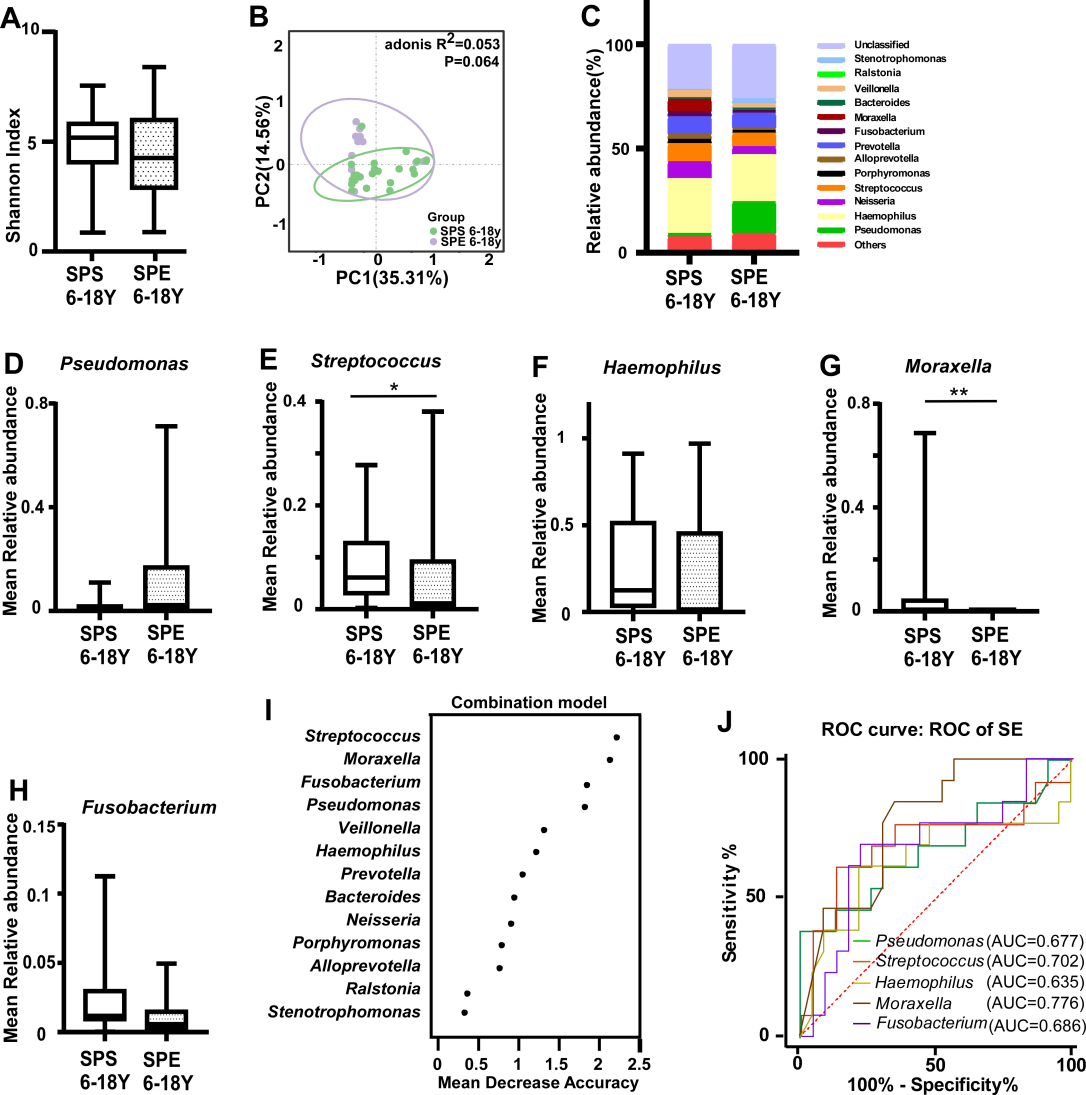
Structural shift in the sputum microbiota in pediatric PCD patients aged 6–18 years. (A) Microbial diversity among the SAS and SAE groups aged 6–18 years (Shannon index; *P* > 0.05, *n*1: *n*2 = 23:13). (B) Principal coordinate analysis (PCoA) based on weighted UniFrac distance by analysis of similarities (Adonis) in the SAS and SAE patients aged 6–18 years (*P* < 0.05). (C) The relative abundance of microbial taxa at the genus level in the SAS and SAE groups aged 6–18 years; genera with a relative abundance ranking in the top 1%. (D–H) Median relative abundance of *Pseudomonas* (D), *Streptococcus* (E), *Haemophilus* (F), *Moraxella* (G), and *Fusobacterium* (H) in patients aged 6–18 years in SPS and SPE groups. (I) The relative abundance of microbial taxa at the genus level. Classification performance of the top 1% most discriminant genera of the SPE patients aged 6–18 years by a random forest model. (J) Receiver operating characteristic (ROC) curves and their corresponding area under the curve (AUC), employing the top five genera in the SPE patients aged 6–18 years. Boxes show the 25th–75th interquartile range (IQR). The median is indicated by a solid line in the box. PCD: primary ciliary dyskinesia, SPS: sputum of pediatric stable stage, SPE: sputum of pediatric exacerbation stage, *: *P* < 0.05; **: *P* < 0.01 for statistically significant differences.

The dominant microbiota in pediatric PCD patients was different from that in adults, so we further explored the relationship between patients of different ages with pulmonary exacerbation and the sputum microbiota. The mean age at diagnosis was 8.3 years, and FEV1 declined at 6 years (20), so it is important to determine the factors of pulmonary exacerbation in earlier stages. As there were only two sputum samples in the pulmonary exacerbation group from patients aged <6 years, we analyzed the sputum microbiota correlation in the pulmonary exacerbation group aged 6–18 years. There was no significant difference in the Shannon diversity index or PERMANOVA between the two groups (Fig. 2A and B); however, specific microbiota exhibited distinct relative abundances between the groups (Fig. 2C). The relative abundance of *Pseudomonas* in sputum of pediatric exacerbation stage patients aged 6–18 years was higher, while that of *Haemophilus, Streptococcus*, *Moraxella*, and *Fusobacterium* was lower (Fig. 2D through H); only *Streptococcus* and *Moraxella* had significant differences. According to the random forest analysis mentioned above, *Streptococcus* and *Moraxella*, which were among the top five genera, exhibited moderate distinguishing effects in the pulmonary exacerbation group, with area under the curve (AUC) values of 0.702 and 0.776 in the discovery cohort (Fig. 2I and J).

### Differences between BALF and sputum microbiota in children

**Fig 3:**
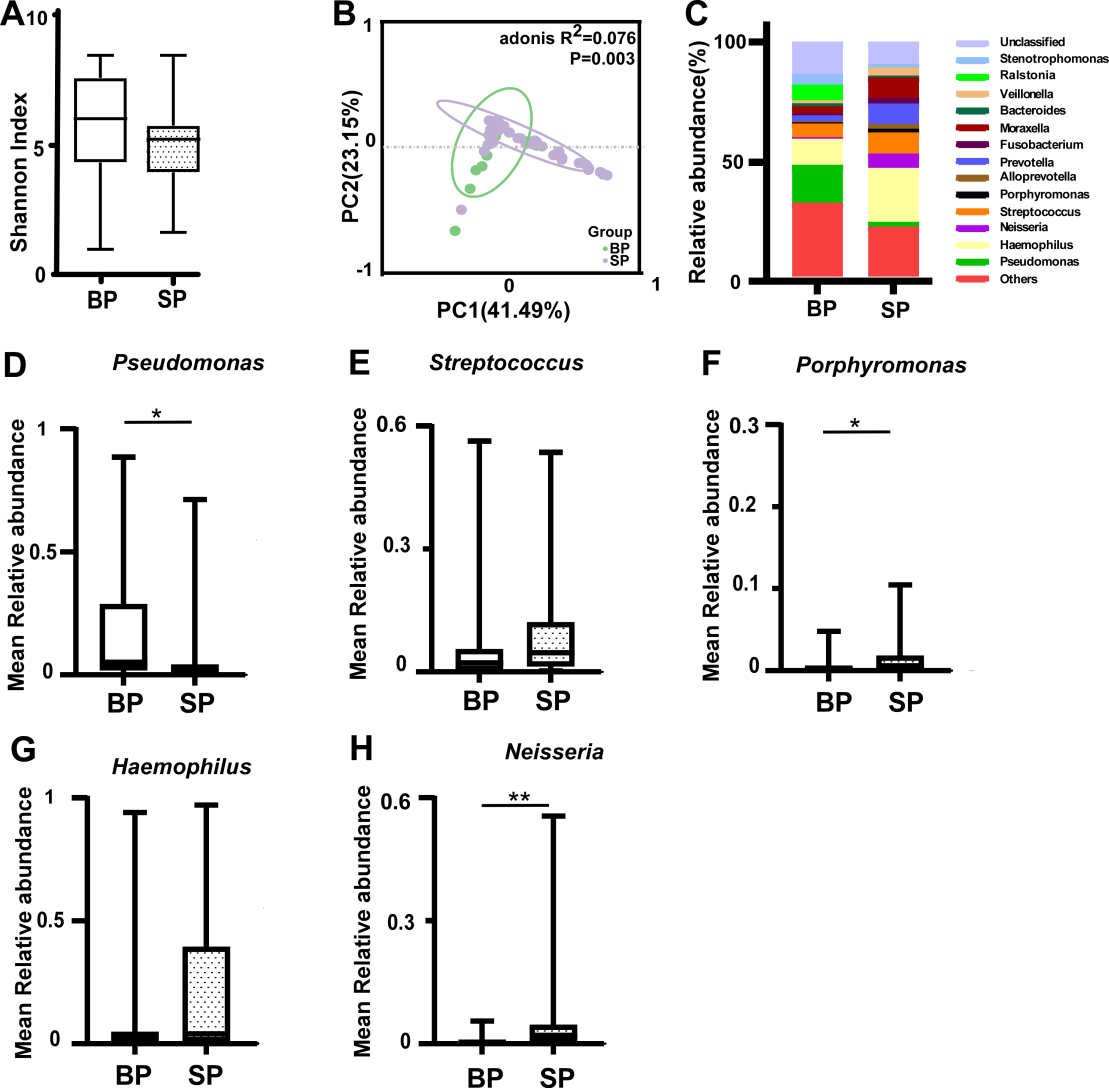
Comparison of the BALF and sputum microbiota in children with PCD. (A) Microbial diversity in the BP and SP groups (Shannon index; *P* > 0.05). (B) Principal coordinate analysis (PCoA) based on weighted UniFrac distance by analysis of similarities (Adonis; *P* < 0.01). (C) The relative abundance of microbial taxa at the genus level; genera with a relative abundance ranking in the top 1%. (D–H) Median relative abundances of *Pseudomonas* (D), *Streptococcus* (E), *Porphyromonas* (F), *Haemophilus* (G), and *Neisseria* (H) in the BP and SP groups. Boxes show the 25th–75th interquartile range (IQR). The median is indicated by a solid line in the box. PCD: primary ciliary dyskinesia, BP: pediatric bronchoalveolar lavage fluid (*n* = 17), SP: pediatric sputum (*n* = 49), *: *P* < 0.05; **: *P* < 0.01 for statistically significant differences.

Bronchoscopy with BALF collection can be utilized to identify pathogens, but it is not recommended for routine surveillance in PCD patients because it requires sedation and has not been shown to improve outcomes (21, 22). BALF represents the real pathogeny but was rarely obtained. The Shannon index of pediatric sputum (SP) was lower than that of pediatric BALF (BP); however, they were not significantly different (Fig. 3A). The SP group had a significant difference in PCoA compared with the BP group (PERMANOVA: *P* = 0.003; *R* ^2^ = 0.076; Fig. 3B). Furthermore, we found increased microbiota compositional dissimilarity in the sputum group, implying a more complex heterogeneous community structure compared with the BP group (Fig. 3C). At the genus level, the relative abundance of *Pseudomonas* in the BP group was significantly higher than that in the SP group (Fig. 3D); those of *Streptococcus*, *Porphyromonas*, *Haemophilus*, and *Neisseria* were lower, but only *Porphyromonas* and *Neisseria* had significant differences (Fig. 3E through H).

### BALF microbiota dominated by *Pseudomonas* are associated with pulmonary exacerbation

**Fig 4:**
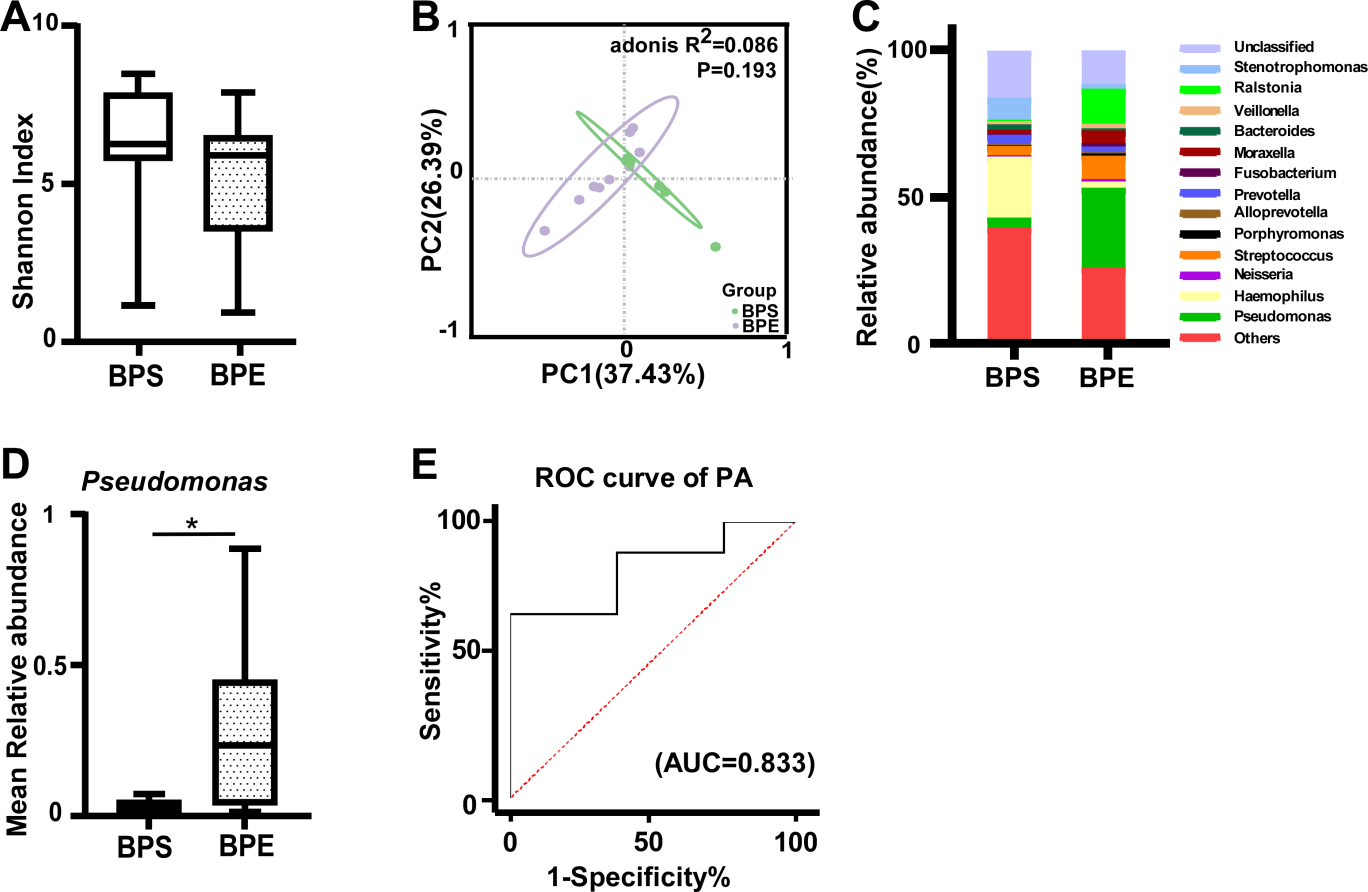
Structural shift in the BALF microbiota in children with PCD with pulmonary exacerbation. (A) Microbial diversity among the BPS and BPE groups (Shannon index; *P* > 0.05). (B) Principal coordinate analysis (PCoA) based on weighted UniFrac distance by analysis of similarities (Adonis; *P* = 0.193). (C) The relative abundance of microbial taxa at the genus level; genera with a relative abundance ranking in the top 1%. (D) Median relative abundance of *Pseudomonas* associated with BPS and BPE PCD patients. (E) Receiver operating characteristic (ROC) curves and their corresponding area under the curve (AUC) for *Pseudomonas* in the BPS and BPE groups. Boxes show the 25th–75th interquartile range (IQR). The median is indicated by a solid line in the box. PCD: primary ciliary dyskinesia, BALF: bronchoalveolar lavage fluid, BPS: pediatric stable bronchoalveolar lavage fluid (*n* = 8), and BPE: bronchoalveolar lavage fluid of pediatric exacerbation (*n* = 9), *: *P* < 0.05; **: *P* < 0.01 for statistically significant differences.

To precisely distinguish the different microbiota categories and richness between pulmonary exacerbation and pulmonary stabilization, we compared the 16S sequences in pediatric stable BALF (BPS) samples with those in BALF samples of pediatric exacerbation (BPE). First, the Shannon index in the BPE group was lower than that in the BPS group (6.260 vs. 5.904, *P* = 0.178); however, no significant difference was found between the groups (Fig. 4A). Second, PCoA presented a difference in the microbiota diversity between the two groups, but there was no significant difference due to the limited sample size (Fig. 4B). Third, at the genus level, there was increased microbiota compositional dissimilarity in the BPE group compared with the BPS group. Moreover, the BPE group was characterized by a higher abundance of *Pseudomonas*, *Streptococcus*, *Porphyromonas*, *Fusobacterium*, *Moraxella*, and *Ralstonia* and a lower abundance of *Haemophilus* (Fig. 4C); however, only *Pseudomonas* was significantly different between the two groups (Fig. 4D). *Pseudomonas* had a moderate discriminating effect for BPE, with an AUC of 0.833 and a sensitivity of 66.67% (Fig. 4E).

## DISCUSSION

In this large single-center study of PCD microbiota, we created an outline of the bacterial microbes in the respiratory tract; furthermore, the correlation with pulmonary exacerbation was compared. We found that the microbiota diversity in SP was richer than that in pediatric BALF through sequencing, indicating a heterogeneous community structure. The microbiota beta diversity and richness were lower during pulmonary exacerbation than during pulmonary stabilization, but compositional dissimilarity was reduced because the dominant genus was highly abundant. A significantly higher abundance of *Pseudomonas* had a moderate distinguishing effect for lung exacerbation.

Pulmonary exacerbation in PCD patients is caused by persistent lung infections, chronic inflammation, and changes in the lower airway microbiota over time, which leads to significant morbidity in patients with this condition (23, 24). According to previous reports, *Pseudomonas* is the most common bacterial pathogen in adults, while *Haemophilus* is the most common pathogen in children (6, 25, 26), which is consistent with our findings. There was no consensus reached about the microbiota associated with pulmonary exacerbation. In our research, we found significant differences in *Pseudomonas*, *Streptococcus*, and *Haemophilus* in pulmonary exacerbation, which can be used to distinguish exacerbation by random forest analysis. However, among the top 1% of genera, only *Pseudomonas* had a moderate discriminating effect in pediatric pulmonary exacerbation, owing to being more sensitive than traditional culture. The SAE group had a higher prevalence of *Pseudomonas* infection (27), but no significant difference was found between the SAS and SAE groups, owing to the small sample size.

In children with acute respiratory tract infections (RTIs), the viral and bacterial communities in paired nasopharyngeal and tracheal samples showed considerable overlap, indicating that the upper respiratory tract microbiota could be the origin of the lower respiratory tract microbiota (28). The most common cultured genera were *Pseudomonas*, *Haemophilus*, *and Moraxella*, regardless of sputum or BALF, which was consistent with other studies (7, 26). However, sequencing can be used to detect some gram-negative bacilli, such as *Stenotrophomonas*, *Bacillus*, *Ralstonia*, and *Escherichia-Shigella*, in BALF. Although *Ralstonia* has been identified in PCD and CF patients in the past, its role in PCD is poorly understood (13, 26, 29). The prevalence of *Ralstonia* infection in CF patients remains unclear due to the challenges in isolating the organism; Fluit and colleagues reported the identification of only nine *Ralstonia* isolates out of a total of 286 specimens, indicating a low prevalence of *Ralstonia* infection in CF patients (30). Similar to *Ralstonia* leading to the loss of microbial diversity in severe lung disease (31), it is probably harmful in PCD patients. *Stenotrophomonas* is increasingly prevalent among individuals with CF and is considered one of the most common emerging multidrug-resistant organisms found in the lungs (32). In addition, one study showed that its emergence is associated with a loss of microbial diversity and severe lung disease in individuals with CF (33). *Stenotrophomonas* in PCD patients may also be harmful, although related research is rare. The results indicate that a complementary strategy involving both traditional culture-based methods and advanced molecular techniques could enhance microbiological diagnostics in the future, although further investigations are warranted to elucidate the clinical significance of positive sequencing results in the context of negative cultures.

Some longitudinal cohort studies have shown a correlation between the early development of respiratory microbiota and susceptibility to RTIs as well as its impact on respiratory health later in life (34, 35). This study demonstrated that the relative abundance of *Pseudomonas* was significantly higher during pulmonary exacerbation in teenagers and adults than in preschool children, which was consistent with the age trend in previous reports (6). A recent study found that the *Pseudomonas* interactome indicates that interaction networks are linked to exacerbation risk and that including microbial interaction data enhances clinical prediction models (11). In this study, we found that *Pseudomonas* had a moderate predictive effect on pulmonary exacerbation, and we can use appropriate antibiotics when considering *Pseudomonas* infection in preschool PCD patients. Previous studies found that *Haemophilus* and *Streptococcus* were increased in early childhood (6), but *Haemophilus* may have some uncertain influence on clinical features in preschool children, perhaps associated with decreased pulmonary function. *Streptococcus* was among the top three genera in children; its abundance decreased with age and had a distinguishing effect on pulmonary exacerbation. All this evidence highlights the contribution of *Streptococcus*, particularly in early childhood, in PCD patients, which should attract more research attention. *Prevotella* is typically considered a normal commensal microbe in the upper respiratory tract in healthy individuals. However, it has been related to increased levels of IL-17+ CD + T cells and neutrophils, indicating an immune response in lower RTIs (36). *Prevotella* detected by culture may influence airway inflammation in CF patients (10), and it may be associated with airway inflammation and repeated infection in adult PCD patients. *Staphylococcus* is one of the dominant genera in adults (7), but its relative abundance was not in the top 20, perhaps due to its strong pathogenicity.

Our study has some limitations. The administration of antibiotics when collecting samples was found to be correlated with reduced microbial diversity, but antibiotic type and duration were not clear because most patients had already received different antibiotic treatments at the time of consultation (37). Due to the small sample size, there were some changes in the Shannon index and relative abundance of taxa among different groups, but the differences were not statistically significant. Our study assessed BALF only from pediatric patients, and since we are a children’s hospital, we were able to acquire only a small number of sputum samples from adults. 16S rRNA sequencing has limitations in precisely identifying species and cannot be used to detect viruses or fungi. If necessary, metagenomic sequencing can be used for a more comprehensive analysis. We were unable to provide pulmonary function data for children under 6 years of age due to limitations in the detection technology.

### Conclusion

In this study of PCD respiratory tract microbiology by 16S sequencing, we found that the beta diversity and richness of the microbiota were decreased during pulmonary exacerbation. Pulmonary exacerbation correlated positively with *Pseudomonas* and negatively with *Streptococcus*, *Haemophilus*, *Moraxella*, and *Veillonella. Pseudomonas* had a moderate distinguishing effect on lung exacerbation, and we should pay more attention to *Pseudomonas* infection in preschool PCD patients.

## Data Availability

The raw data from 16S sequencing have been deposited in the NCBI Sequence Read Archive (SRA) database (http://www.ncbi.nlm.nih.gov/sra) with the accession number PRJNA1008078. All data needed to evaluate the conclusions in this paper are presented in the paper and the Supplementary Materials. Additional data are available from the authors upon request.

## SUPPLEMENTAL MATERIAL

Table S1 spectrum.02213-23-s0001.xlsx Detailed summary characteristics of the study population.

Table S2 spectrum.02213-23-s0002.xlsx Shannon index of the study population.

Table S3 spectrum.02213-23-s0003.xlsx Top 1% genus relative abundance of the study population.

ASM does not own the copyrights to Supplemental Material that may be linked to, or accessed through, an article. The authors have granted ASM a non-exclusive, world-wide license to publish the Supplemental Material files. Please contact the corresponding author directly for reuse.
